# Acute effects of whey protein, alone and mixed with other macronutrients, on blood pressure and heart rate in older men

**DOI:** 10.1186/s12877-022-03213-1

**Published:** 2022-06-28

**Authors:** Avneet Oberoi, Caroline Giezenaar, Kylie Lange, Karen L. Jones, Michael Horowitz, Ian Chapman, Stijn Soenen

**Affiliations:** 1 Adelaide Medical School and Centre of Research Excellence in Translating Nutritional Science to Good Health, The University of Adelaide, Royal Adelaide Hospital, AdelaideSouth-Australia, Australia; 2 Riddett Institute, Massey University, Palmerston North, 9430 New Zealand; 3 Faculty of Health Sciences and Medicine, Bond University, Robina, QLD 4226 Australia

**Keywords:** Aging, Diet, Whey protein, Blood pressure, Heart rate

## Abstract

**Background:**

Caloric supplements are increasingly used by older people, aiming to increase their daily protein intake. These high caloric drinks, rich in glucose and whey-protein in particular, may result in potential harmful decreases in blood pressure (BP). The effect of ingesting whey-protein with glucose and fat on BP is unknown. It has also been assumed that the maximum fall in systolic blood pressure occurs within 2 h of a meal.

**Methods:**

This study aimed to determine in older men, the effects of whey-protein, alone and mixed with other macronutrients, on systolic (SBP) and diastolic (DBP) blood pressure and heart rate (HR) in older men for 3 h. Thirteen older men (age 75 ± 2yrs; body mass index (BMI) 25.6 ± 0.6 kg/m^2^) ingested a drink on separate study days: (i) 70 g whey-protein (P_280_)_;_ (ii) 14 g whey-protein, 28 g carbohydrate, 12.4 g fat (M_280_); (iii) 70 g whey-protein, 28 g carbohydrate, 12.4 g fat (M_504_); or (iv) a non-caloric control drink (C).

**Results:**

SBP decreased after all three nutrient drinks compared to the C, with the greatest reduction after the M_504_ drink (*P* = 0.008). Maximal decreases in SBP (C: -14 ± 2 mmHg, P_280_: -22 ± 2 mmHg, M_280_: -22 ± 4 mmHg, M_504_: -24 ± 3 mmHg) occurred about 2 h after drink ingestion and this fall was sustained thereafter (120-180 min: P_280_ and M_504_ vs. C *P* < 0.05). Maximum DBP decreases and HR increases occurred after M_504_, with no differences between the effects of the P_280_ and M_280_ drinks.

**Conclusions:**

The effects of whey-protein containing drinks to lower BP and increase HR appear to be primarily dependent on their energy content rather than macronutrient composition and may persist for at least 3 h after ingestion,. Pure whey-protein drinks may represent the best approach to maximize protein intake without increasing the potential for deleterious BP falls in older people.

**Trial registration:**

ACTRN12614000846628, 14/03/2019.

## Introduction

Older people are increasingly encouraged to take high protein nutritional supplements to reduce the age-associated loss of muscle mass and function [1]. Whey protein is often part of these supplements, given that it is high in essential amino acids and appears to be effective in stimulating muscle protein formation [2].

Ingestion of nutrients can lead to postprandial reductions in blood pressure (BP), in older people (even when apparently healthy) [3, 4], in part, caused by postprandial diversion of blood to the gut, which can lead to syncope, falls and, in some cases, stroke or death [5–7]. This so-called postprandial hypotension (PPH) has been defined as a fall in systolic blood pressure (SBP) greater than 20 mmHg during the 2 h following nutrient ingestion i.e. it is assumed that the maximum fall in SBP occurs within 2 h. We have recently reported, in a cohort of healthy older men, that ingestion of a 70 g (280 kcal) whey-protein drink decreased SBP substantially when compared to a non-caloric control drink [8]. In the majority of men in that study magnitude of the SBP decrease was greater than 20 mmHg after the 70 g whey protein drink (11/19 compared to 5/19 after the control drink) [5]. Furthermore, the hypotensive effect of a whey protein drink was prolonged, with a sustained reduction in SBP being evident at 3 h after ingestion. It has been suggested that hypertensive men may be of particular risk of blood pressure falls following food intake [9]. The current study aimed to determine in older men the effects of whey protein, when ingested in a lower quantity but with carbohydrate and fat as occurs frequently in a “real world” setting, on blood pressure and heart rate for 3 h. We hypothesized that the hyportensive effects of whey protein containing drinks would be dependent on the energy rather than protein content of the drink and often persist for more than 2 h.

## Materials and methods

### Subjects

Thirteen older men were recruited by advertisement. Subject characteristics are detailed in Table 1

**Table 1:** Subjects characteristics

Age (years)	75 ± 2
Height (m)	1.75 ± 0.01
Weight (kg)	79 ± 2
BMI (kg/m^2^)	25.6 ± 0.6

Mean and standard error of mean of 13 older men

Exclusion criteria were smoking; being vegetarian; alcohol intake of > 2 standard drinks on > 5 days per week;; use of prescribed or non-prescribed medications which may affect appetite, body weight, gastrointestinal function or energy metabolism; intake of any illicit substance; known lactose intolerance or food allergy(s); epilepsy; gallbladder, pancreatic, cardiovascular or respiratory diseases; significant gastrointestinal symptoms including as abdominal pain, gastro-esophageal reflux, diarrhea, or constipation or surgery; any other illness deemed significant by the investigator; low levels of plasma ferritin; blood donation in the previous 12 weeks; undernutrition (score < 24 on the Mini Nutritional Assessment [10]); depression (score ≥ 11 on the Geriatric Depression Questionnaire [11]); impaired cognitive function (score < 25 on Mini Mental State [12]); or inability to comprehend the study. Anti-hypertensive medication were taken by four older men (; anti-arrhythmic *n* = 1; angiotensin-converting enzyme inhibitor, *n* = 1; beta blockers, *n* = 1; angiotensin receptor blockers *n* = 1). Participants were instructed to not take medication on the morning of their study visit.

The study was conducted in accordance with the Declaration of Helsinki and Royal Adelaide Hospital Human Research Ethics Committee approved the protocol. The study was registered with the Australian New Zealand Clinical Trial Registry (www.anzctr.org.au, registration number ACTRN12614000846628). All subjects provided written informed consent prior to their study inclusion.

### Protocol

Each participant was studied on four occasions in a randomised, double-blind, placebo-controlled design (using randomly permuted blocks; www.randomization.com), separated by 3–14 days, to determine the effects of drinks (~ 450 mL) containing either: (i) 70 g whey protein (280 kcal; ‘P_280_’); (ii) 14 g whey protein, 28 g carbohydrate, 12.4 g fat (280 kcal; ‘M_280_’); (iii) 70 g whey protein, 28 g carbohydrate, 12.4 g fat (504 kcal; ‘M_504_’); or (iv) an iso-palatable control drink (~ 2 kcal; ‘control’) on SBP, DBP and heart rate (HR). The BP and HR data are secondary outcomes of a published study which included results relating to energy intake, appetite, gastric emptying and plasma gut hormone concentrations [13]. Sample size and power calculations for the original study were based on the primary outcomes of energy intake and gastric emptying [13].

The drinks were prepared by homogenizing olive oil (Bertolli Australia Pty Ltd., Unilever Australasia, Sydney, NSW, Australia) and dissolving whey protein isolate (Fonterra Co-Operative Group Ltd., Palmerston North, New Zealand) and dextrose, in varying volumes of demineralized water and diet lime cordial (Bickford’s Australia Pty Ltd., Salisbury South, SA, Australia), to achieve the desired composition, on the morning of the study day, by a research officer, who was not involved in the data analysis. The drinks were stirred continuously at low speed on a stirring plate to ensure even mixing, were matched for taste and served in a covered cup. Both the investigator and the subject were blinded to the drink condition.

Subjects were provided with a standardized meal [beef lasagne (McCain Foods Pty Ltd, Wendouree, Victoria (VIC), Australia), ∼591 kcal] to consume on the night before each study day at ∼1900 h. They were instructed to fast overnight ~ 12 h from solids and liquids except water, and to refrain from strenuous physical activity until they attended the laboratory at ∼0830 h.. The recruitment of participants started in March 2014, and the study was completed in December 2014.

### Measurements

On arrival to the clinical research facility lab, level 4 at Adelaide Health and Medical Sciences, The University of Adelaide, Australia, subjects were seated in an upright position where they remained throughout the study. An intravenous cannula was inserted for blood sampling. Subjects sat quietly for 15 min then had 3 baseline measures of BP (Dinamap machine) and heart rate at 3-min intervals before drinking the test drink within 2 min. BP and HR were measured every 3 min until t = 180 min.. Participants did not receive any other drink or food throughout the study.

### Data and statistical analysis

Statistical analyses were performed using SPSS software (version 24; IBM, Armonk, NY). Drink-condition effects (control, P_280_, M_280_, M_504_) were determined by using two-way repeated-measures ANOVA. Significant effects were followed by Bonferroni corrected post-hoc tests to determine which specific drink conditions were different. Statistical significance was accepted at *P* < 0.05. All data are presented as means ± SEMs. Baseline blood pressure represented as BL in the figures was calculated as an average of -9, -6 and -3 min readings. T = 0 min refers to the point immediately after drink consumption.

## Results

The study protocol was well tolerated by all subjects. No subject reported symptoms of dizziness, faintness or any other adverse events during the study.

### Systolic Blood Pressure (SBP)

Baseline SBP values were not different on the four study days (Mean control: 128 ± 3 mmHg, P_280_: 129 ± 3 mmHg, M_280_: 130 ± 5 mmHg, M_504_ 127 ± 4 mmHg, *P* = 0.95).

**Fig. 1:**
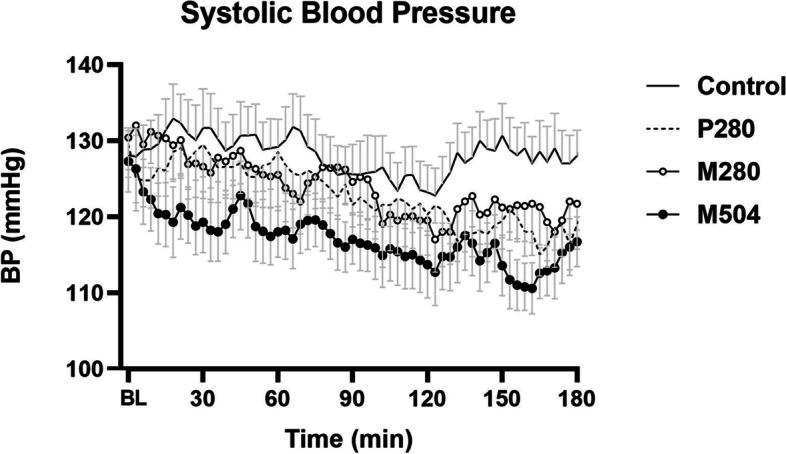
Mean (± SEM) systolic blood pressure (SBP; mmHg) following drink ingestion containing (i) flavored water (control, ~ 2 kcal) or (ii) 70 g whey protein (280 kcal; ‘P_280_’); (iii) 14 g whey protein, 28 g carbohydrate, 12.4 g fat (280 kcal; ‘M280’); (iv) 70 g protein, 28 g carbohydrate, 12.4 g fat (504 kcal; ‘M_504_’) in older (*n* = 13) men. Drink-condition effects were determined by using repeated-measures ANOVA. Baseline blood pressure represented as BL in the figure was calculated as an average of -9, -6 and -3 min readings. SBP was lower after the M_504_ drink when compared to control (*P* = 0.019) during the second (60-120 min *P* = 0.035) and third (120-180 min *P* = 0.005) hour

SBP did not change over the three hours after the control drink. SBP was lower after all three nutrient drinks compared to the control drink, particularly during the second (60-120 min *P* = 0.018) and third (120-180 min *P* = 0.022) hours, with the greatest reduction after the M_504_ drink (*P* = 0.008) (Fig. 1). SBP following M_504_ ingestion was lower when compared to the P_28_0 drink between 0–60 min (*P* = 0.044). There was no significant difference between SBP readings after the P_280_ and M_280_ drinks (*P* > 0.05).

Following drink ingestion, a decrease in SBP > 20 mmHg occurred at some time in 3/13 for control, 7/13 for P_280_, 6/13 for M_280_, and 9/13 for M_504_. Maximal SBP decreases from baseline (control: -14 ± 2 mmHg, P_280_: -22 ± 2 mmHg, M_280_: -22 ± 4 mmHg, M_540_ -24 ± 3 mmHg; *P* = 0.11) occurred about two hours after the drinks (time baseline to nadir SBP: control: 99 ± 16 min, P_280_: 119 ± 13 min, M_280_: 116 ± 12 min, M_504_: 119 ± 15 min; *P* = 0.86) and was sustained thereafter following the nutrient drinks (average 120-180 min control: 128 ± 3 mmHg, P_280_: 118 ± 2 mmHg, M_280_: 120 ± 4 mmHg, M_504_:114 ± 3 mmHg).

### Diastolic Blood Pressure (DBP)

**Fig. 2:**
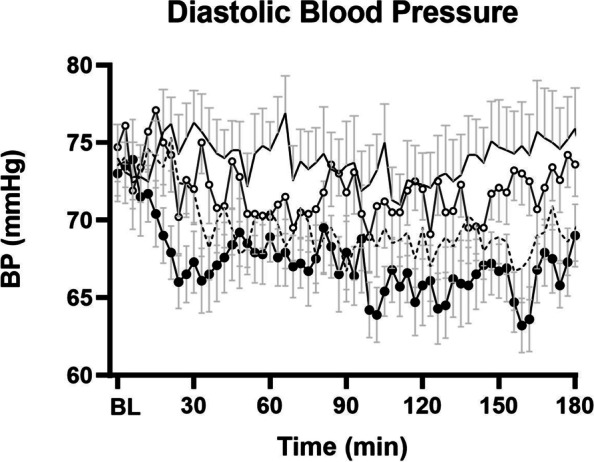
Mean (± SEM) systolic blood pressure (DBP; mmHg) following drink ingestion containing (i) flavored water (control, ~ 2 kcal) or (ii) 70 g whey protein (280 kcal; ‘P_280_’); (iii) 14 g whey protein, 28 g carbohydrate, 12.4 g fat (280 kcal; ‘M_280_’); (iv) 70 g protein, 28 g carbohydrate, 12.4 g fat (504 kcal; ‘M_504_’) in older (*n* = 13) men. Drink-condition effects were determined by using repeated-measures ANOVA. Baseline blood pressure represented as BL in the figure was calculated as an average of -9, -6 and -3 min readings. DBP was lower after the M_504_ (*P* < 0.001) and P_280_ (*P* = 0.018) drinks when compared to control during the second (*P* = 0.012) and third (*P* = 0.035) hour. There was no statistically significant difference between the effects of M_280_ and P_28_0 on BP

Baseline DBP values were not different on the four study days (Mean control: 74 ± 2 mmHg, P_280_: 74 ± 2 mmHg, M_280_: 75 ± 3 mmHg, M_504_ 73 ± 2 mmHg, *P* = 0.94). DBP was lower after all three nutrient drinks compared to the control drink, with the greatest reduction after M_504_ (Fig. 2).

DBP did not change over the three hours after the control drink. DBP was less after M_504_ (*P* < 0.001) and P_280_ (*P* = 0.026) when compared to control – the drink-condition effect was significant during all three hours following drink ingestion (0–60 min *P* = 0.002, 60-120 min *P* = 0.004, 120-180 min *P* = 0.003). There was no difference between the effects of M_280_ and P_280_ on DBP (*P* > 0.05).

Maximal DBP decreases from baseline (control: -12 ± 2 mmHg, P_280_: -15 ± 1 mmHg, M_280_: -15 ± 2 mmHg, M_504_: -15 ± 1 mmHg, P = 0.17) occurred on average between one to two hours after drink ingestion (time baseline to nadir: control: 80 ± 17 min, P_280_: 108 ± 16 min, M_280_: 71 ± 12 min, M_504_: 99 ± 15 min, *P* = 0.24) and were sustained during the third hour for P_280_ and M_504_ (average control: 74 ± 1 mmHg, P_280_: 68 ± 1 mmHg, M_280_: 71 ± 1 mmHg, M_504_: 66 ± 1 mmHg).

### Heart Rate (HR)

Baseline HR values were not different on the four study days (Mean control: 58 ± 2 bpm, P_280_: 59 ± 3 bpm, M_280_: 59 ± 2 bpm, M_504_ 59 ± 3 bpm, *P* = 0.95).

**Fig. 3:**
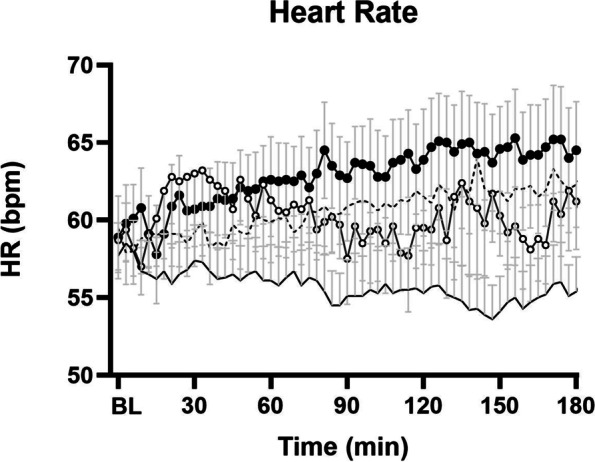
Mean (± SEM) Heart Rate (HR; bpm) following drink ingestion containing (i) flavored water (control, ~ 2 kcal) or (ii) 70 g whey protein (280 kcal; ‘P_280_’); (iii) 14 g whey protein, 28 g carbohydrate, 12.4 g fat (280 kcal; ‘M_280_’); (iv) 70 g protein, 28 g carbohydrate, 12.4 g fat (504 kcal; ‘M_504_’) in older (n = 13) men. Drink-condition effects were determined by using repeated-measures ANOVA. Baseline Heart rate represented as BL in the figure was calculated as an average of -9, -6 and -3 min readings. HR increased after the M_504_ (*P* < 0.001) and P_280_ (*P* = 0.017) drinks when compared to control (0-180 min)

HR decreased over 3 h after the control drink and increased after the M_504_ (*P* < 0.001) when compared to control, and did not change significantly after either the M_280_ or P_280_ drinks (0-180 min average control: 57 ± 1 bpm, P_280_: 60 ± 1 bpm, M_280_: 60 ± 2 bpm, M_504_: 63 ± 1 bpm; Fig. 3). The drink-condition effect was significant during all the three hours (0–60 min *P* = 0.001, 60-120 min *P* < 0.001, 120-180 min *P* < 0.001) hour following drink ingestion.

Maximal HR increase from baseline (control: 3.8 ± 1 bpm, P_280_: 9 ± 2 bpm, M_280_: 12 ± 4 bpm, M_504_ 13 ± 2 bpm, *P* < 0.001.) occurred on average between one to two hours after drink ingestion (time baseline to peak: control: 48 ± 14 min, P_280_: 116 ± 15 min, M_280_: 83 ± 14 min, M_504_: 127 ± 16 min, *P* = 0.003) and were sustained during the third hour for P_280_ and M_504_ (average control: 55 ± 1 bpm, P_280_: 61 ± 1 bpm, M_280_: 60 ± 1 bpm, M_504_:64 ± 1 bpm).

## Discussion

The major observation in this study is that in healthy older men following ingestion of nutrient drinks containing 280 or 504 kcal energy as pure whey protein or as mixed macronutrients, the magnitude of the decrease in BP is dependent on the energy content, rather than, the protein content of the drinks. Furthermore, while the onset of the BP reduction was evident soon after nutrient drink ingestion, the hypotensive effect was sustained for at least 3 h –. Both observations are of clinical relevance in a ‘real world’ setting. There is no advantage as far as the risk of postprandial hypotension by consuming protein with other macronutrients.

This is also consistent with the context that the rate of gastric emptying of nutrients, whether carbohydrate, fat or protein is primarily dependent on their caloric content.

Heart rate increased after ingestion of the nutrient drinks compared to the control drink, with the greatest and more sustained increase after the highest energy load drink. The resultant increase in cardiac output represents a compensatory mechanism for the potential postprandial fall in BP caused by diversion of blood to the gut. The nutrient-induced increases in HR were inadequate, however, to compensate fully for this diversion in these older men. We have reported smaller increases in HR and larger decreases in BP in healthy older than younger men after a pure 70 g whey protein drink [8] indicating that an age-related reduction in the ability to increase the heart rate after food ingestion contributes to the greater reduction in BP observed in older than younger adults after nutrient ingestion.

The nutrient drink-induced reduction in BP were substantial- a decrease of 20 mmHg SBP or more occurred at some time in 77% of the 504-kcal drink days, compared to 31% of the control drink days. None of the participants reported symptoms of dizziness or faintness, but they were seated throughout the study and not permitted to get up and walk around. A fall in systolic BP of 20 mmHg or more is clearly e associated with symptoms and an increased likelihood of falls and injury, as reflected in one definition of postprandial hypotension [5]. It is not known whether the risk of symptoms is influenced by the baseline systolic pressure and this should be evaluated. However, it should be recommended that current criteria for definition of hypertension do not take into account the relationship of the blood pressure measurement to meal ingestion. Our observations suggest that caution should possibly be exercised for several hours (certainly more than two) at least in older people mobilising after nutrient containing drinks in relation to the risks of syncope and falls,. Individuals at risk may potentially benefit from more frequent, lower calorie drinks or meals. Further studies should evaluate this strategy, [14]. The fall in BP may also be dependent on whether food is already present in the stomach and/or small intestine at the time a nutrient supplement is ingested.

Limitations of this study include that it was only conducted in men. While women tend to have a lower baseline BP that would intuitively predispose them lower BP levels after a meal, we and others have shown that the magnitude of the postprandial fall in BP is related directly to baseline BP such that the fall is greater in those who are hypertensive [9, 15] Further limitations include that the participants remained sitting to standardize study conditions and the number of participants was relatively small. Nevertheless, the observed decreases in BP and HR were clear cut. Because, the effects of the drinks on BP were still evident at 3 h, when each study ceased, it would be of relevance to determine the total duration of the postprandial hypotensive effect. Our study was designed to clarify phenomenology of ‘real world’ relevance rather than mechanisms. In relation to the later, evaluation of autonomic function would be of interest. We also only evaluated the acute effects of drinks, although we have no reason to believe that their effects on BP will be modified by chronic use.

Our observations suggest that if the intention is to give an older person a nutritional supplement drink containing as much whey protein as possible to preserve, or potentially enhance, muscle mass and function, administering it in as pure a form as possible (i.e., with the minimum amount of fat and carbohydrate) may potentially reduce the BP the least and so minimize the risk of postprandial hypotension. If, on the other hand, the intention is to provide a mixed macronutrient supplement to provide a specific amount of energy, it should be appreciated the total energy content of the supplement, rather than its macronutrient composition will be the major determinant of its hypotensive effect.

## Conclusions

The hypotensive effect of mixed macronutrient drinks is dependent on overall energy intake rather than macronutrient composition of a drink and may be sustained for at least 3 h. Pure whey protein drinks may, accordingly represent the best approach to maximise protein intake while minimising the potential for deleterious BP falls in older people.

## Data Availability

The datasets generated and/or analyzed during the current study are not publicly available due to ethical restrictions of the protocol having mentioned in our approved local ethical application that data will not be available for the general public but are available from the corresponding author on reasonable request.

## Abbreviations

BMI: Body mass index
SEM: Standard error of mean
SBP: Systolic blood pressure
DBP: Diastolic blood pressure
HR: Heart rate
